# COVID-19 Is a Multifaceted Challenging Pandemic Which Needs Urgent Public Health Interventions

**DOI:** 10.3390/microorganisms8081228

**Published:** 2020-08-12

**Authors:** Carlo Contini, Elisabetta Caselli, Fernanda Martini, Martina Maritati, Elena Torreggiani, Silva Seraceni, Fortunato Vesce, Paolo Perri, Leonzio Rizzo, Mauro Tognon

**Affiliations:** 1 Infectious Diseases and Dermatology Section, Pathology, Oncology and Experimental Biology Section, Department of Medical Sciences, University of Ferrara, 44124 Ferrara, Italy; fernanda.martini@unife.it (F.M.); trrlne@unife.it (E.T.); tgm@unife.it (M.T.); 2 Department of Chemical and Pharmaceutical Sciences, Microbiology Section, University of Ferrara, 44124 Ferrara, Italy; elisabetta.caselli@unife.it; 3 Department of Morphology, Surgery and Experimental Medicine, University of Ferrara, 44124 Ferrara, Italy; mrtmtn@unife.it; 4 RDI, Rete Diagnostica Italiana s.r.l, Lifebrain Group, Limena, 35010 Padova, Italy; silva.seraceni@lifebrain.it; 5 Department of Morphology, Surgery and Experimental Medicine, Obstetrics and Gynecology Section, University of Ferrara, 44124 Ferrara, Italy; fortunato.vesce@unife.it; 6 Department of Biomedical Sciences and Surgical Specialties; Ophthalmology Section, University of Ferrara, 44124 Ferrara, Italy; paolo.perri@unife.it; 7 Department of Economy and Management; University of Ferrara, 44124 Ferrara, Italy; leonzio.rizzo@unife.it

**Keywords:** SARS-CoV-2, COVID-19, cytokine storm syndrome, COVID-19 planet, hyperinflammation, endothelitis, thromboembolism

## Abstract

Until less than two decades ago, all known human coronaviruses (CoV) caused diseases so mild that they did not stimulate further advanced CoV research. In 2002 and following years, the scenario changed dramatically with the advent of the new more pathogenic CoVs, including Severe Acute Respiratory Syndome (SARS-CoV-1), Middle Eastern respiratory syndrome (MERS)-CoV, and the new zoonotic SARS-CoV-2, likely originated from bat species and responsible for the present coronavirus disease (COVID-19), which to date has caused 15,581,007 confirmed cases and 635,173 deaths in 208 countries, including Italy. SARS-CoV-2 transmission is mainly airborne via droplets generated by symptomatic patients, and possibly asymptomatic individuals during incubation of the disease, although for the latter, there are no certain data yet. However, research on asymptomatic viral infection is currently ongoing worldwide to elucidate the real prevalence and mortality of the disease. From a clinical point of view, COVID-19 would be defined as “COVID Planet “ because it presents as a multifaceted disease, due to the large number of organs and tissues infected by the virus. Overall, based on the available published data, 80.9% of patients infected by SARS-CoV-2 develop a mild disease/infection, 13.8% severe pneumonia, 4.7% respiratory failure, septic shock, or multi-organ failure, and 3% of these cases are fatal, but mortality parameter is highly variable in different countries. Clinically, SARS-CoV-2 causes severe primary interstitial viral pneumonia and a “cytokine storm syndrome”, characterized by a severe and fatal uncontrolled systemic inflammatory response triggered by the activation of interleukin 6 (IL-6) with development of endothelitis and generalized thrombosis that can lead to organ failure and death. Risk factors include advanced age and comorbidities including hypertension, diabetes, and cardiovascular disease. Virus entry occurs via binding the angiotensin-converting enzyme 2 (ACE2) receptor present in almost all tissues and organs through the Spike (S) protein. Currently, SARS-CoV-2 infection is prevented by the use of masks, social distancing, and improved hand hygiene measures. This review summarizes the current knowledge on the main biological and clinical features of the SARS-CoV-2 pandemic, also focusing on the principal measures taken in some Italian regions to face the emergency and on the most important treatments used to manage the COVID-19 pandemic.

## 1. Background

Human coronaviruses (CoVs) are members of the subfamily Coronavirinae from the family Coronaviridae and the order Nidovirales. CoVs are a group of highly diverse, enveloped, positive-sense, single-stranded RNA enveloped viruses (+ssRNA) that range from 26 to 32 kilobases. The name coronavirus derives from the crown-shaped tips that are present on their surface [1]. They are divided into four genera, including α-/β-/γ-/δ-CoV. α- and β-CoV able to infect mammals, while γ- and δ-CoV mainly infect birds, whereas seldom infect mammals including rodents and bats [1,2]. Previously, six CoVs have been identified as human-susceptible viruses, among which α-CoVs, CoV-229E, CoV-NL63, β-CoVs, CoV-HKU1, and CoV-OC43 own low pathogenicity, cause mild respiratory symptoms similar to a common cold, respectively [3]. 

Transmission of CoVs is usually via airborne droplets to the nasal mucosa in closed environments and through close contact between people, unwashed non-sanitized hands, and rarely as a result of touching contaminated surfaces. No other route of transmission of coronavirus infection has been documented thus far. The virus replicates locally in cells of the ciliated epithelium of respiratory tract, causing cell damage and inflammation. Incidence peaks occur in the winter, taking the form of local epidemics lasting few weeks or months. It has been reported that the same serotype may return to an area after several years [2].

## 2. Impact of Coronaviruses in Animals and Humans

CoVs cause a broad spectrum of diseases in domestic and wild animals, including camels rodents, birds, rabbits, ferrets, minks, snakes, bats, and poultry, ranging from mild to severe enteric, respiratory, and systemic disease [1]. 

In temperate climates, respiratory CoV infections occur more often in the winter and spring than in the summer and fall. CoV infections have shown to contribute as much as 35% of the total respiratory viral activity during epidemics. The predominant illness associated with CoVs is an upper respiratory infection with occasional cases of pneumonia in infants and young adults [1,4]. CoVs have also been shown to be effective in producing asthma exacerbations in children, as well as chronic bronchitis in adults and the elderly [5]. The milder forms caused by the four strains (OC43, NL63, HKU1, and 229E) of CoVs are found globally and are responsible about one-fifth of common colds or flu without leaving permanent immunity [4]. 

The ancestral origins of major CoVs including 229E strain likely involve African bat hosts as demonstrated by their finding in fecal specimens from Ghana [6]. Bats are consumed throughout Africa as wild meat [7]. Humans often live near bat caves, use bat guano as fertilizer, and drink water from these caves. These settings potentially facilitate the exposure of humans and their domestic animals, including camelids, to these CoVs from bats [7].

The common cold is due to a viral infection of the upper respiratory tract. A total of more than 200 cold-associated viruses are known, and often more than one viral species is involved in the infection at the same time. The most commonly responsible virus is rhinovirus (30–80% of cases), a genus of Picornaviridae with 99 known serotypes; other viral aetiological infectious agents include coronavirus (10–15%) and Orthomyxoviridae (5–15%), human parainfluenza virus, human respiratory syncytial virus, adenovirus, enterovirus, and metapneumovirus. Besides, similar symptoms can also be caused by bacterial infections (Table 1).

Many CoV infections are subclinical [11,12]. There is speculation about the association of CoV with serious human diseases, such as multiple sclerosis [13], hepatitis, or enteric diseases [14] in infants. However, none of these early associations has been conclusively demonstrated. On the contrary, immunocompromised children may be predisposed to severe CoV infections [1]. Nevertheless, until less than two decades ago, all known human varieties of CoV caused diseases so mild that they did not stimulate further advanced coronavirus research. The scenario has changed dramatically in the current century, in which every decade has been characterized by the advent of a new and more pathogenic CoV.

## 3. Emergent Zoonotic Coronaviruses of the Century Responsible for Coronavirus Epidemics and Pandemics 

In the last twenty years, three new highly pathogenic zoonotic β-CoVs, which caused lethal human diseases and generated great media clamor and public concern, have emerged: (i) the SARS CoV (now called SARS-CoV-1) discovered in November 2002 [12], (ii) the Middle Eastern Respiratory Syndrome (MERS) CoV (MERS-CoV), first identified in Saudi Arabia in June 2012 [15], and (iii) SARS-CoV-2, formerly named 2019-nCoV when it was identified in December 2019 after the sequencing of respiratory clinical samples from patients in Wuhan, Hubei province, China [16]. The disease caused by SARS-CoV-2 is now called Coronavirus Diseases-2019 (COVID-19).

Interestingly, SARS and MERS zoonotic CoVs have probably originated from bats and then moved into other mammalian hosts—the Himalayan palm civet for SARS-CoV and the dromedary camel for MERS-CoV—before spilling over humans [17,18,19]. The SARS epidemic was the first human pandemic to break out in the 21^st^ century and emerged through recombination of bat SARS-related coronaviruses [20]. Transmission of SARS-CoV-1 occurred from the bat to humans and the palm civets served as a secondary host and reservoir for the continuation of the infection and then the SARS emergency [21]. The SARS epidemic, which started in 2002 and ended in 2003, lasted about 1 year and has so far caused 8098 cases in 26 countries in 2002–2003, with 774 deaths (estimated mortality rate of 10%). No other cases have been detected since 2004. Uncertainty remains as to the epidemiology and ecology of the infection. The virus reservoir has been detected in bats, but the passage to humans took place through an intermediate host, civet, which is considered a food delicacy in China. The main mechanism of morbidity and lethality of SARS is the so-called acute respiratory distress syndrome (ARDS). The virus-cell interaction occurs between the viral Spike protein, the ligand, and the human angiotensin-converting enzyme 2 (ACE2), which is the membrane cellular receptor. ACE2 is a key player enzyme in the Renin-Angiotensin system and a target for the treatment of hypertension [22]. The Spike/ACE2 interaction triggers a violent acute inflammation of the lungs, leading to the formation of a layer of fibrin on the pulmonary alveoli, thus preventing gaseous exchange [23]. The transmission of SARS-CoV-1 occurred mainly person-to-person through direct contact, Flügge’s large drop-to-drop contact, and indirect contact by fomites, unwashed hands, and rarely touching contaminated surfaces or air travel [24,25]. No effective drugs have been identified, although vaccination strategies have been designed to fight SARS pandemic [26].

MERS (Middle East Respiratory Syndrome) originated in 2012 in Saudi Arabia and then spread to other countries in the Middle East, where it recorded the highest number of cases, and worldwide (27 countries at the peak of the epidemic), including Europe. 

Unlike SARS-CoV-1, MERS-CoV likely spilled over from bats to dromedary camels at least 30 years ago and since then has been prevalent in dromedary camels [27]. 

MERS-CoV binds to a receptor called dipeptidyl-peptidase 4 (DPP4) also known as CD26 (dipeptidyl-peptidase 4, DPP4), highly expressed in unciliated bronchial epithelial cells and type II pneumocytes from lower bronchi and lung cells in the kidney, alveoli, small intestine, liver, and prostate, and on activated leukocytes [28]. The MERS has never disappeared and the infection continues to be transmitted to humans by camelids, matt, llamas, while man-to-man transmission has mainly affected health care workers who have treated patients [29]. Even in January 2020, sporadic cases were reported in the United Arab Emirates. The total cumulative number of cases is 2499, with 861 deaths (estimated lethality 34%).

Although the natural reservoir identifies the dromedaries as the intermediate host, the exact modalities of species jumping with transmission to humans have not been defined [20,30]. Patients with MERS have, in addition to serious respiratory disorders, important intestinal complications and sometimes acute renal damage [29]. This different clinical spectrum has been related to a different viral input receptor, the CD26 molecule, expressed not only by the epithelium of the lower respiratory tract but also by the intestinal and renal tract [29]. As for SARS, neither effective specific drugs nor vaccines have been developed for MERS, and measures to contain and prevent secondary transmission are limited.

## 4. New SARS-CoV-2 Insights

SARS-CoV-2 arose from Wuhan, Hubei Province, China. It spread rapidly through all the world’s countries and to date has killed 635,173 persons with more than 15,581,009 cases of in the world, including Europe, in which currently there have been over 208,580 deaths. The number of infected cases is steadily increasing especially in the USA, South America (Brazil), South East Asia, and Africa [16].

Its origin is still discussed, although the initial cases have been associated with the Huanan South China Seafood Market. However, there is no absolute evidence so far that the origin of SARS-CoV-2 was from the seafood market [31]. 

In Europe, the first few cases have been reported in France and Germany and then in Italy and Spain. In France, the COVID-19 epidemic is believed to have started between the end of December 2019 and late January 2020 [32]. Here, it was not found a link with China, and the lack of recent foreign travel suggests that the disease was already spreading among the French population at the end of December 2019 [32]. In Italy, the first cases of CoV 2019-nCoV infection were diagnosed at the end of January in a couple of Chinese tourists from Wuhan Province, when they arrived on 23 January [33]. Bayesian phylogenetic reconstruction suggested that these two tourists were probably infected before their arrival in Italy, since the viral strains sequences intermixed with Chinese isolates of the epidemic dating back probably to 19 January, 2020 before their arrival in Italy. Furthermore, the Chinese tourist’s viral strains isolated in Italy clustered with other European strains from France and Germany, intermixing with other Chinese sequences, thus suggesting that strains introduced in Europe to date came from China [18].

The results are consistent with several introductions of SARS-CoV-2 in Europe and/or further circulation of the single strain originating in Wuhan with concurrent evolution and accumulation of mutations. Further studies will be needed to assess the actual occurrence of SARS-CoV-2 in Europe and the extent of SARS-CoV-2 contamination in the European population between the end of 2019 and January 2020 and to explore potential unnoticed deaths that may have occurred at that time [18].

As happened with SARS-CoV-1 and MERS-CoV, SARS-CoV-2 almost certainly originated in bats of the species horseshoe bat (*Rhinolophus hipposideros* and *Rhinolophus affinis*) and adapted to non-bat ACE2 receptor variants as it crossed species to infect humans. The most recent analysis of the SARS-CoV-2 genome has found that this virus shares 96 percent of its RNA with a coronavirus that was previously identified in a specific bat species in China, suggesting that bat CoV and human SARS-CoV-2 might share the same ancestor [17,34]. 

On the basis of recent phylogenetic data, it is not unlikely that SARS-CoV-2 passed directly from bats to humans without the intermediate host. Moreover, it cannot be excluded that for SARS-CoV-2 the spillover effect may have involved a mechanism similar to those found in SARS or MERS, although an intermediate host was not found. However, there may be other animals including snakes, turtles [35], and pangolins that would serve as intermediate hosts, as demonstrated by protein sequence alignment and phylogenetic analysis [18,35,36,37,38,39,40]. Pangolins, long-snouted, ant-eating mammals, were suggested as the possible intermediate hosts because coronavirus genomes from pangolins had approximately 85.5–92.4% similarity to SARS-CoV-2, representing two sub-lineages of SARS-CoV-2 in the phylogenetic tree, one of which (GD/P1L and GDP2S) was extremely closely related to 2019-nCoV [36]. However, even if the pangolin may not be the intermediate host for SARS-CoV-2, the possibility of viral recombination of SARS-CoV-2 in pangolin cannot be excluded [35].

Compared with the known SARS-CoV-1 and MERS-CoV genome, SARS-CoV-2 shows homology of 92.83%, while on the amino acid sequence of the S protein, the homology is even 97.25% (Table 2). Currently, the main source of infection is the patient with SARS-CoV-2. Patients with non-clinically evident disease [41] or incubation [42] or convalescent [43] may also be a source of infection. There are two main routes of infection: respiratory droplets and close contact. The possibility of aerosol transmission in a relatively closed environment exists. The existence that superspreaders may exist in the early stage has been postulated [44]. Generally, the mouth, nose, and ocular mucosa appear to be the major way of transmission. In the eye, ACE2 receptor is not expressed in the conjunctival or corneal epithelium but is expressed in the retinal and retinal pigment epithelium [45,46]. SARS-CoV-2 may enter the tears through droplets and then be transferred to the respiratory tract through the nasolacrimal canal. Indeed, conjunctivitis is one of the symptoms of COVID-19, which is often indistinguishable from other viral conjunctivitis. Although further studies are needed, the risk of transmission of SARS-CoV-2 through tears is low, even if the virus was demonstrated in tears or conjunctival secretions of symptomatic patients [47]. In addition, it has not been demonstrated that there is a correlation between the presence of the virus in the nasopharyngeal swab (NPS), oropharyngeal swab (OPS), and ocular secretion [47].

The fecal-oral transmission route remains to be determined, although SARS-CoV-2 exists in feces and rectal swab specimens of infected patients [48,49]. SARS-CoV-2 RNA may persist in these patients even after the disappearance of respiratory symptoms and when NPS/OPS are negative [50]. Some data also suggest that transmission via the digestive tract may be a potential transmission route for the virus based on the ACE2 receptor study of SARS-CoV-2 [11,51]. Recently, the fecal specimen was highly recommended for routine detection of SARS-CoV-2 and especially before discharging COVID-19 patients [52].

Recently, evidence of vertical transmission has been reported [53]. Although pregnant women are constitutively less at risk of COVID infections, as well as SARS and MERS, probably due to genetic and host factors, in most women who have had signs of mild to moderate COVID-19 pneumonia, no loss of pregnancy and premature birth occurred [54]. Recent findings also suggest that there have been no confirmed cases of intrauterine transmission of SARS-CoV-2 from mothers with COVID-19 to their fetuses and placenta, which were negative for RT-PCR for SARS-CoV-2 [55]. However, the neonatal diagnosis of SARS-CoV-2 should not be limited to molecular testing, when there is also the possibility of cultivating the virus in vitro. In fact, a recent Italian author highlighted the importance of viral culture to be used in parallel with molecular techniques to detect the presence of cytopathogenic viral agents, as demonstrated in an Italian 7-week-old lactating infant who tested positive for SARS-CoV-2 only with the cell culture method, without any clinical suspicion and/or risk factor for SARS-CoV-2 infection [56]. More detailed studies will be required to confirm these preliminary results. SARS-CoV-2 can survive in the environment from a few hours to a few days, depending on surfaces and environmental conditions, and touching affected surfaces, such as mobile phone and paper money or where the virus is presumed to survive for up to 2 days [57].

According to the Centers for Disease Control and Prevention (https://www.cdc.gov/coronavirus/2019-nCoV/index.html), whether a person can acquire COVID-19 by touching surfaces or objects contaminated with the virus, then touching mucosal membranes, remains to be confirmed [58].

## 5. The Mechanism of SARS-CoV-2 Entry 

Previous analysis of SARS-CoV-2 strongly suggests that this new CoV, like SARS, uses ACE2 receptor, a target for the treatment of hypertension [22], to gain entry to cells. Accumulating data suggest that the lung is the most important and vulnerable target organ of the SARS-CoV-2. It is possible that the large lung surface area makes the lung highly susceptible to inhaled viruses. Indeed, 83% of the cells expressing human ACE2 receptor are type II alveolar epithelial cells, which can serve as a reservoir for viral invasion, promoting rapid viral expansion and a vicious circle of destruction of the local alveolar wall. This loop results in a rapidly progressive and widespread alveolar damage [11]. Transmembrane Protease, Serine 2 (TMPRSS2) by contrast, is a transmembrane serine protease receptor (required for S protein priming, which may prevent cell entry of SARS-CoV-2 [59]. A TMPRSS2 inhibitor blocking entry might constitute a treatment option of SARS-CoV-2 [60]. Thus, SARS-CoV-2 cell entry depends on surface molecules such as ACE2 and TMPRSS2.

SARS-CoV-2 binds to ACE2 through the S-glycoprotein comprising two subunits, S1 and S2 [11], which are involved in virus-host and cell tropism (S1) and fusion of the virus-cell membrane (S2), respectively. Other than the S protein responsible for facilitating entry of virus into the target cell, the coronavirus genome encodes three structural proteins: nucleocapsid (N), membrane (M), and the envelope (E) polypeptides [35]. SARS-CoV-2 recognizes human ACE2 receptor more efficiently than SARS-CoV-1; this may increase the ability of SARS-CoV-2 to be transmitted from person to person, warranting a more efficient spread among subjects [61].

**Figure 1:**
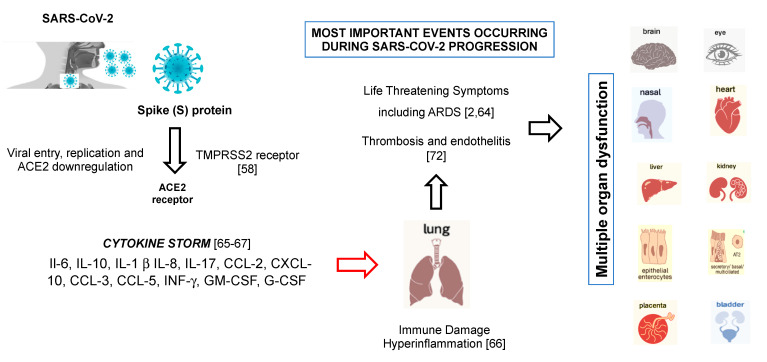
Angiotensin-converting enzyme 2 (ACE2) expression is also found in respiratory and pulmonary tract cells (alveolar monocytes and macrophages), with the possibility of severe acute respiratory distress syndrome (ARDS) and in heart, kidneys, brain, endothelium, liver, in which organ failure and thromboembolism may occur.

After membrane fusion (Figure 1), the viral RNA is released into the cytoplasm, facilitating its replication. The abundant expression of the ACE2 receptor in type II alveolar cells promotes a rapid viral multiplication with the subsequent destruction of the local alveolar wall. This process results with a rapidly progressive and widespread alveolar damage with the occurrence of concomitant cytokine/chemokine cascades (Figure 1). In this setting, females seem to have fewer ACE2 receptors on the lung surface than males and this could contribute, together with some genetic and hormonal factors, to give more protection to COVID-19 infection and make them less susceptible [62]. Studies are underway to support these hypotheses.

Recent studies have demonstrated the virus entry mechanism, also clarifying the contribution of such mechanism to the evasiveness of the virus against immune response, cell infectivity, and spreadability of the virus [63]. Basically, differently from other CoV, SARS-CoV-2 entry is dependent on preactivation by proprotein convertase furin, thus reducing its need for target cell proteases. Such a mechanism allows SARS-CoV-2 to maintain efficient cell entry while evading immune surveillance, thus potentially contributing to the wide spread of the virus. 

ACE2 receptor is also highly expressed on the luminal surface of intestinal epithelial cells, which may explain some initial disease manifestations, such as vomiting and diarrhea [64]. Thus, the intestine might be a major entry site for SARS-CoV-2. However, less than 10% of children with infection develop diarrhea and vomiting [65]. The infection of human gut epithelium has important implications for fecal–oral transmission and containment of viral spread. Whether COVID-19 may have started with the consumption of bat food from the Wuhan market, the presumed location of the outbreak, is yet to be determined [11]. Moreover, ACE2 receptor tissue distribution in organs such as heart, kidney, endothelium, and retina could explain the multi-organ dysfunction observed in patients.

## 6. SARS-CoV-2 and the Cytokine Storm Syndrome

Accumulating evidence suggests that SARS-CoV-2 causes an inflammatory response in the lower airway, leading to lung injury (Figure 1). Collectively, the virions first invade the respiratory mucosa and then trigger a powerful immune response in the lungs with the production of a cytokine storm (Cytokine Storm Syndrome; CSS) that hyperactivates a type-1 cellular T-helper response similar to that described in SARS and MERS [31,66]. CSS is an uncontrolled and often fatal systemic inflammatory response, resulting from the release of large amounts of pro-inflammatory cytokines, including IFN-α, IFN-γ, IL-1β, IL-6, IL-12, IL-17, IL-18, IL-33, TNF-α, TGFβ, and chemokines, including CCL2, CCL3, CCL5, CXCL8, CXCL9, and CXCL10. Cytokines/chemokines all contribute to the occurrence of ARDS and may lead to the most critical condition of COVID-19 patients, including death [31,67]. 

While the main target of CoVs is lung epithelial cells, SARS-CoV-2 also infects macrophages and dendritic cells, which overstimulated and promote hyperinflammation [68]. Most cytokines produced during SARS-CoV are secreted by macrophages and other mononuclear phagocytes. Therefore, macrophages could be a key target to stop the extensive lung damage caused by COVID-19 [67]. IL-17 is able to strengthen the inflammation response and to activate neutrophils cells that can migrate to the lung and are heavily involved in the pathogenesis of SARS-CoV-2 infection. Blocking IL-17 could provide a novel therapeutic strategy for COVID-19 [69].

CSS is an extremely life-threatening situation that can lead to harmful effects, such as loss of capillaries, tissue toxicity, organ failure, and shock. The activation of platelets triggered by this cytokine activation can contribute to hypercoagulability, the cause of pulmonary thromboembolism [66] leading to death from respiratory failure (Figure 1).

Since the ACE2 receptor is also widely distributed in organs, such as heart, kidney, endothelium, and retina, this could explain the multi-organ dysfunction observed in COVID-19 affected patients. Therapeutic studies based on the rational use of anti-inflammatory drugs that directly inhibit the synthesis processes of inflammatory cytokines, e.g., inflammasome Nucleotide binding oligomerization domain (NOD) NOD-like receptor family, pyrin domain containing 3 (NLRP3) [68], are currently underway in these patients. Early control of CSS by immunomodulators and cytokine antagonists at an early stage, as well as reduction of lung inflammatory cell infiltration, is the key to improving treatment success rates and reducing mortality rates in patients with COVID-19. Therefore, rational therapy should always include a careful evaluation of the cytokine’s profile of selected cohorts of symptomatic COVID-19 patients, especially those with severe pneumonia and in intensive care units (ICU).

## 7. Clinical Manifestations: The “COVID-19 Planet” 

Most clinical trials originally came from China, whereas they were mainly focused on lung damage leading to death in COVID-positive patients from severe respiratory failure. Upon SARS-CoV-2 arrived in Europe and around the world, the virus was found also to target many other organs. Therefore, from a clinical point of view, COVID-19 would be defined as the “COVID Planet” because of the multifaceted clinical and laboratory aspects of the disease, related to the large number of tissues involved by SARS-CoV-2 [70]. The initial stages of COVID-19 infection are nonspecific and include symptoms typical of multiple respiratory illnesses (Table 1). Most cases are mild (81%), being the symptoms usually self-limiting with the recovery occurring in two weeks [71]. In fact, on the basis of existing Chinese data published so far, 80.9% of patients infected with the virus develop mild infection and include symptoms nonspecific and typical of multiple respiratory illnesses, viral and bacterial. Some of these, which may occur simultaneously with COVID-19, should be considered in the differential diagnosis (Table 1). In agreement with Chinese studies, patients develop severe pneumonia in 13.8% of cases, respiratory failure in 4.7%, and septic shock or even multi-organ failure in 5%. Of these, 3% are fatal [31,70,71,72]. The cause of death among infected patients is severe respiratory failure. Patients with severe presentation, closely resembles SARS-CoV-1 and/or MERS-CoV infections [73], may develop ARDS and require ICU admission, although oxygen therapy and assisted intubation may not be useful because of pulmonary and generalized venous thromboembolism [66,74]. 

**Figure 2:**
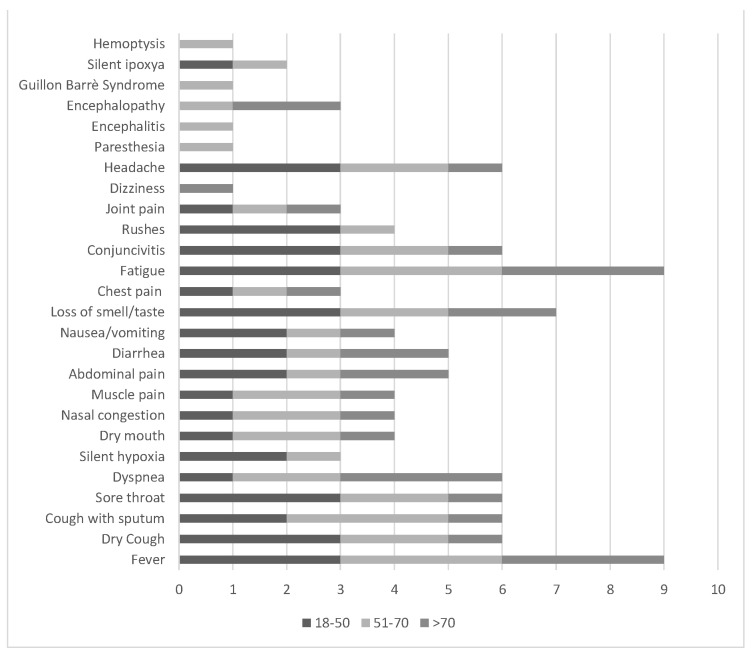
Frequency of COVID-19 clinical features divided into age groups (scale: 0–3; 0 = not observed, 3 = frequently observed).

The range age of hospitalized patients is 57–79 years, with 1/3–1/2 affected by an underlying illness. In general, there is a higher risk of death for males at 2.8%, while it remains at 1.7% for females [19,75]. Available data suggest that COVID-19 has an incubation period of ~5–7 days (range 2–14 days). The complete clinical profile of COVID-19 is not fully understood. Symptoms may appear in as few as 2 days or after as many as 14 (estimated ranges vary from 2–10 days, 2–14 days, and 10–14 days), during which the virus is contagious, but the patient does not display any symptom (asymptomatic transmission). The most common symptoms [19,48,68,69] are fever, cough, pharyngitis, dyspnea, rarely kidney failure, suggesting fever is dominant, but not the main symptom of infection (Figure 2). A small number of patients can have headache/hemoptysis/diarrhea and even be relatively asymptomatic [42,48]. Patients with chronic underlying diseases, including hypertension, cardio-cerebrovascular diseases, and diabetes or other conditions that may compromise their immune systems, may increase the risk of SARS-CoV-2 infection [48]. Smoking may be a negative prognostic indicator for COVID-19 [48]. As for SARS, no deaths have been reported in the pediatric age group, especially under the age of 10 years old [76]. If in adults the virus triggers an abnormal and disproportionate immune system reaction in critical cases, in children the immune response is more balanced towards infection and there is often an optimal recovery within a week, as respiratory involvement, when present, tends to be more benign [31].

A number of less frequent symptoms were described during COVID-19, suggesting that SARS-CoV-2 does not remain confined to the respiratory tract. These symptoms, often underestimated, are the feeling of weakness and fatigue, sore throat, stuffy nose, headache, myalgia, asthenia, chills, vomiting, and nausea [70]. The central nervous system may be affected with headache, nausea, vomiting, instability, ideomotor slowdown, ataxia, epilepsy; the peripheral nervous system with hypogeusia, hyposmia, and neuralgia and the skeletal muscle system may also be affected [31,70,77,78]. Moreover, cases of encephalopathy, encephalitis, necrotizing hemorrhagic encephalopathy, stroke, epileptic seizures, rhabdomyolysis, and Guillain-Barre syndrome have been reported to be associated with SARS-CoV-2 infection [44,79]. The neuroinvasive propensity of SARS-CoV-2 has, therefore, already been documented in SARS and MERS studies [20,29]. 

Cardiovascular disease appears to be a multiplier of the risk of death in case of COVID-19 especially in hypertensive and COVID-19 positive diabetic patients who are more at risk of complications, such as myocardial infarction, heart failure, myocarditis, arrhythmias, and adverse outcomes [80].

Regarding laboratory findings, most patients have shown lymphopenia, elevated C-reactive protein and erythrocyte sedimentation rate, and decreased oxygenation index. Moreover, elevated levels of liver function, renal function, and D-dimer (25.9%) also observed. Only a few patients had leukocytosis and elevated procalcitonin [31,48,70,77].

## 8. Diagnosis of SARS-CoV-2 Infection 

SARS-Cov-2 infection diagnosis is mostly performed by the reverse transcription polymerase chain reaction (RT-PCR) assay for viral RNA detection, according to the US Centers for Disease Control and Prevention instructions. Although this is the most popular approach, other methods include the CRISPR-based DETECTR assay [81], which provides a faster alternative with 95% positive and 100% negative predictive agreement, point-of-care testing using Specific High Sensitivity Enzymatic Reporter UnLOCKing (SHERLOCK) diagnostics [82], and reverse-transcription loop-mediated isothermal amplification (RT-LAMP) [83]. 

NPS/OPS technique has some limitations, including low sensitivity and the long period of time to obtain the result, which can be very critical for an infected individual/patient. A study from the Johns Hopkins University found that of the NPS/OPS performed on the fifth day after infection, 38% were falsely negative, which drops to 20% on the eighth day, the one recommended to perform the test. After day 8 (3 days after symptom onset) falsely negative results begin to increase again, from 21% (CI, 13% to 31%) on day 9 to 66% (CI, 54% to 77%) on day 21 [84]. 

This limitation also underlies the difficulty of providing accurate real-time information on the evolution of the epidemic. In this regard, testing of specimens from multiple sites may improve the sensitivity and reduce false-negative test results. In this regard, samples from sputum or bronchial lavage or the mucus/catarrh excreted from the lower airway are thought to be more accurate than NPS/OPS [51]. Two smaller studies reported the presence of SARS-CoV-2 in anal or oral swabs and blood from 16 patients in Hubei Province, and viral load in throat swabs and sputum from 17 confirmed cases [51]. It should be considered that a single swab “photographs” a precise moment of the presence of the virus and if a symptomatic subject or subject with few symptoms is negative, the test must be repeated after at least 24/48 h.

Following the identification of COVID-19, the WHO published a list of protocols used by public health and specialist research labs for identification of SARS-CoV-2 [85]. In addition, also due to shortcomings of RT-PCR approach to detect SARS-CoV-2, the serum immunoglobulin (Ig) anti-SARS-CoV-2 type M (IgM)/IgG antibody detection against the SARS-CoV-2 internal nucleoprotein (NP) and surface spike protein receptor-binding domain (RBD) may be used in diagnosis of COVID-19 and to monitor the disease stages and identify past infection and immunity [83,86].

So far, it remains to be determined the value of serological assays for the detection of human immunoglobulin (Ig) anti-SARS-CoV-2 type M (IgM) or type G (IgG) in the diagnosis and monitoring of COVID-19, especially for patients with acute infection. Currently, these tests have limitations as diagnostic tools [87].

## 9. The Fight Against COVID-19 in Northern Italy

**Figure 3:**
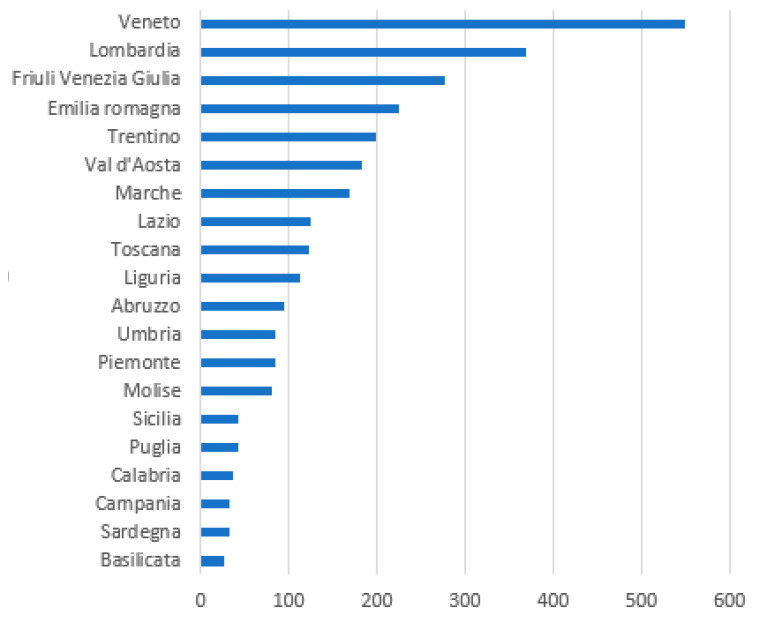
Number of oropharyngeal swab (OPS)/nasopharyngeal swab (NPS) per 1,000,000 inhabitants in Italian regions (period March–April 2020) *Elaborazione GIMBE dati Protezione Civile*—https://coronavirus.gimbe.org/ [88].

The reactions of health systems to COVID-19 in the world differed from country to country. When COVID-19 reached Italy, the first region to be affected was Lombardy, with over 11 million inhabitants. Later, the contagion spread to Veneto and Emilia Romagna and then to all the other Italian regions. Since the beginning of the pandemic, more NPS/OPS have been carried out in Veneto than in Lombardy. This policy has shown that performing a high number of NPS/OPS has led to a greater discovery of positive COVID-19 subjects/patients and to isolate them at home in quarantine, with less overcrowding of hospitals and, therefore, fewer deaths (Figure 3). Looking at the numbers of contagions and deaths and make a comparison between Lombardy and Veneto, these are 87,258 and 19,097 against 15,874 and 1878, respectively, in Lombardy and Veneto.

Initially in Italy, NPS/OPS were carried out on symptomatic subjects only. When the infection spread to other Italian regions including Veneto, it became evident that this policy was not enough and swabs were, therefore, also performed on asymptomatic subjects or those with few or mild symptoms. Veneto was the region that first pursued early SARS-CoV-2 detection by searching for the virus even in asymptomatic subjects and imposing self-isolation or quarantine. This proved to be the winning strategy to stem the spread of the wave of infections, in order to prevent the regional health system from being overwhelmed. 

**Figure 4:**
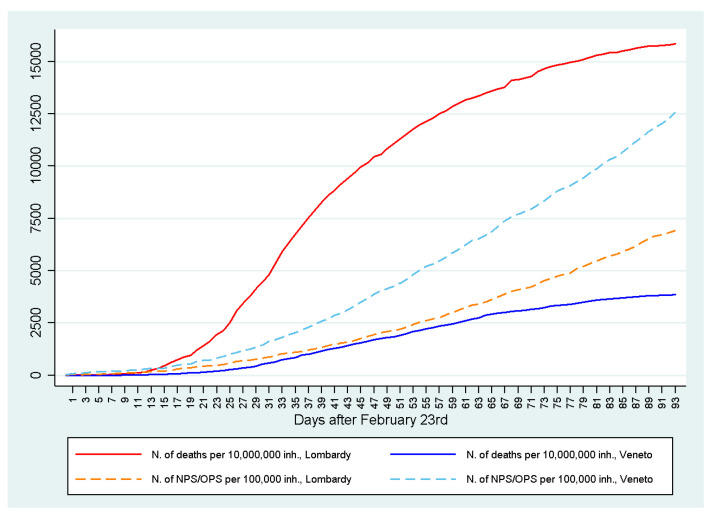
Number of deaths and NPS/OPS in Veneto and Lombardy during the COVID-19 pandemic.

From the data made available by the Italian Civil Protection, it can be seen that since the beginning of the epidemic the number of NPS/OPS carried out in the Veneto region was higher than in Lombardy (Figure 3). This number begins to diverge significantly from the 24th day after the beginning of the epidemic. The Veneto region announced on 17 March that it was going to carry out swabs everyone. In fact, since the following week, swabs have increased much more than in Lombardy. It is important to note that the number of deceased in Lombardy is much higher than in Veneto, and the scissors widens with time and with the increase in the number of swabs in Veneto compared to Lombardy (Figure 4).

Another important finding was that in the small urban town of *Vo’ Euganeo,* where the second outbreak of COVID-19 broke out after Lombardy, it was found that 43.2% of people positive for NPS/OPS were asymptomatic. The experience of the municipality of *Vo’ Euganeo* was, therefore, interesting to analyze because it allowed a closed community to be studied, as the town was quarantined well before the regional and national lockdown that took place three weeks later. Actually, in Veneto and only in this Italian region, there is an epidemiological and biological registry of all the cases, a mapping of the contacts house-by-house, and therefore an ability to act immediately.

Performing more NPS/OPS and analyzing the correlation between NPS/OPS and symptomatic patients and isolated patients at home, the number of NPS per person, carried out ten days earlier, was negatively correlated with the number of people hospitalized with symptoms, while there was a positive correlation with the number of people isolated at home. Specifically, for every thousand more tampons per 100,000 inhabitants, there would be 19 fewer people hospitalized with symptoms and 11 more people isolated at home [89].

Therefore, the numerical increase of NPS/OPS can allow, thanks to the “real photograph” of the virus present in the individual, who has contracted the infection, to have an increase in less clinically seriously patients. Patients with blurred symptoms and a decrease in the more serious ones requiring hospitalization as, if detected early, can be treated at home with anti-inflammatory drugs and heparin, followed through the territorial medicine of the urban district in which they live.

If the pathogenesis of the disease had been known early on and if this policy had been implemented immediately, it would have been possible to prevent the evolution of complications, such as thromboembolism and respiratory failure, thus limiting hospital admissions and potential deaths.

## 10. Conclusions

In only 5 months, after the appearance of SARS-CoV-2, many scientific advances have been made in the understanding of the pathogenesis and the clinical features of this viral disease. 

However, there are various COVID-19 aspects that remain to be elucidated.

1. The lack of knowledge of the exact molecular mechanism of viral entry and replication, which provides the basis for future research into the development of targeted antiviral drugs and vaccines, needs to be filled.

2. We do not know exactly whether SARS-COV-2 mutates and if so, whether these will have an impact on the aggressiveness of the virus in the coming months or whether it will be a more attenuated virus.

3. We still do not know why the male gender is more affected than the female gender. Genetic hormonal differences have been reported, and testosterone seems to favor a more severe evolution of the infection in some cases. In this context, females seem to have fewer ACE2 receptors on the lung surface than males and this could contribute, together with some genetic and hormonal factors, to give more protection to SARS-CoV-2 infection and make them less susceptible [62]. Studies are underway to support these hypotheses.

4. So far, serological assays for the detection of human IgM and IgG currently have limitations in sensitivity/specificity and are not able to provide epidemiological evidence since most of the Italian general population (over 90%) results uninfected by SARS-CoV-2 (unpublished data). Moreover, no completely reliable immunological assays have yet been developed for the detection of serum antibodies against SARS-CoV-2. They probably will be important to define epidemiological questions, including attack rate in the population, and to identify immune individuals. However, it remains to be investigated in detail the immune response elicited by SARS-CoV-2. In addition, it is not known how long the humoral response lasts in the infected subjects.

5. Last but not least. The Veneto experience has revealed that swabs should also be carried out on asymptomatic subjects to control the spread of SARS-CoV-2. In fact, asymptomatic infected subjects may act as a source of infection. The case of municipality of *Vo’ Euganeo*, the only town in the world, has shown that searching and finding for the virus in the completely asymptomatic people of that small village of Veneto of 3000 inhabitants, has allowed to isolate those individuals resulted positive for SARS-CoV-2 and quarantine them, thus avoiding and blocking the spread of the virus. Regarding asymptomatic subjects, a Chinese analysis suggested that the proportion of asymptomatic patients was only around 1%, but recent results suggest that the proportion is closer to 5% [90,91].

Research of asymptomatic infection among swab positive persons is currently ongoing worldwide to elucidate the real prevalence of the disease and the true relative mortality ratio. Recently, no statistically significant difference was found in the viral load of symptomatic versus asymptomatic infections, highlighting the importance of asymptomatic SARS-CoV-2 infection [92].

Moreover, asymptomatic individuals have shown a weaker immune response to SARS-CoV-2 infection. In fact, a recent report showed that the IgG levels and neutralizing antibody levels of the asymptomatic group declined during the early convalescent phase. Therefore, timely RT–PCR and serological testing should be used in conjunction, which would benefit accurate estimation of the asymptomatic proportion [93]. 

## 11. Perspectives

Currently, COVID-19 is only prevented by the use of masks, safety distance, and hand washing. For this disease, there is not yet a vaccine, as was the case for SARS and MERS though several studies are currently underway [94]. To date, there are no medical treatments for COVID-19 with proven effectiveness. Novel treatments and/or vaccines will take time to be developed and distributed to patients. The search for effective treatments for COVID-19 infection is a complex process and not all are recommended in the absence of clear evidence of proven efficacy. SARS-CoV-2 owns potential therapeutic targets similar to those of other RNA CoVs, such as SARS-CoV-1 and MERS-CoV. 

So far, current management often consists of off-label or compassionate therapies, antiparasitic agents including chloroquine, hydroxychloroquine and nitazoxanide, antibiotics, antiretroviral agents, anti-inflammatory and anti-coagulant compounds (heparin and heparin dervatives), and recently, the hyperimmune plasma from recovered patients [10,95]. Several clinical trials are underway to examine existing antiviral drugs and to identify those that could be specific and effective against COVID-19. PubMed, Embase, and Google Scholar were searched to identify articles that supported therapeutic benefit for a pharmacological substance in SARS-CoV-1 or MERS-CoV that could be repositioned for treatment of SARS-CoV-2 infections.

- RNA-Dependent RNA Polymerase Inhibitors (RdRp).
  Among RNA-Dependent RNA Polymerase Inhibitors (RdRp), there is Remdesivir (RDV), an adenosine analog previously used as anti-Ebola virus drug recently approved by Food and Drug Administration (FDA), to employ in emergency for the treatment of COVID-19 in adults and children hospitalized with severe disease [96]. The mechanism of action of RDV makes it potentially useful in the treatment of COVID-19. At least 23 studies of RDV are currently listed on various trial registers, intending to study 23,500 patients, but fewer than a quarter are double-blind, and some are uncontrolled observational studies [97]. Although measurement of efficacy will require ongoing randomized, placebo-controlled trials of RDV therapy, recently, in a cohort of patients hospitalized for severe COVID-19 who were treated with compassionate-use RDV, clinical improvement was observed in 36 of 53 patients (68%) [97]. More recently, our research team proposed to administer RDV by aerosol to combat SARS-CoV-2 in the upper respiratory tract in patients with severe clinical conditions and in ICUs in order to avoid possible side effects of systemic intravenous RDV administration [98]. Other recent potential effective antivirals include Favipiravir (Avigan), in the same class of the RDV, whose antiviral activity is exhibited through selectively targeting RdRp, are attractive targets for antiviral therapies, which interrupt the nucleotide incorporation process during viral RNA replication [90]. Favipiravir is a guanine analog approved for treatment against influenza virus infection in Japan and also can effectively inhibit replication of Ebola, yellow fever, chikungunya, norovirus, and enterovirus and could potentially exhibit effects against SARS-CoV-2 [99]. Neuraminidase and protease inhibitors have not proven to be particularly effective against SARS-CoV-2, although additional studies are necessary.
- Inhibitors of TMPRSS2 serine protease.
  The serine protease TMPRSS2 required for SARS- CoV-2 entry into host cells, highly conserved among the MERS-CoV, SARS-CoV-1, and SARS-CoV-2 viruses, has been identified as a promising target for treatment of COVID-19 [59]. The TMPRSS2 cell entry of SARS-CoV-2 inhibitors camostat mesylate, nafamostat, and bromhexine may treat COVID-19 [60].
- Anti-inflammatory molecules potentially efficacy against SARS-CoV-2.
  For severe COVID-19 illness, many experimental studies are underway to test the validity of anti-inflammatory molecules used successfully for other diseases that may have a potential anti-SARS-CoV-2 effect [100]. In this context, the use of corticosteroids (dexamethasone) as well as the immunomodulators sarilumab, tocilizumab, meplazumab, bevacizumab, baricitinib, avipatadil, may ameliorate the cytokine storm that contributes to mortality [101]. Tocilizumab has been widely used in rheumatic diseases, such as rheumatoid arthritis. It is an IL-6 receptor (IL-6R) blocker that can effectively block the IL-6 signal transduction pathway and thus is likely to become an effective drug for patients with severe COVID-19 and to reduce the mortality [102]. To block IL-17 pathway by biological drugs that are already available and used to treat different pathologies could also be a novel, additional strategy to treat patients infected by SARS-CoV-2 [71]. COVID-19 was initially underestimated especially in Lombardy and other regions of Northern Italy, in which hospitals have become amplifiers of SARS-CoV-2 infection. If these patients were treated early with anti-inflammatory drugs and anticoagulants, they could have avoided being hospitalized, where many of them died.
- Antagonists of Proteinases.
  The growing recognition of endothelitis and thrombosis in COVID-19 patients provides a strong incentive to determine the potential utility of antagonists of PAR1 (Proteinase-activated receptor 1) inhibitors to improve the outcome of such patients. PAR1 is widely expressed in cell types relevant to COVID-19 pathobiology, which include pneumocytes, endothelial cells, fibroblasts, and platelets. Activation of PAR1 by the serine protease thrombin is a critical element in platelet aggregation and coagulation. In particular, serine protease inhibitor Nafamostat, a serine protease inhibitor that works as an anticoagulant, has demonstrated satisfactory results in inhibiting the action of MERS-CoV and has been shown to be effective against SARS-CoV-2 infection, preventing membrane fusion [103].
- *Hyperimmune plasma*.
  The use of hyperimmune plasma obtained from convalescent patients recovered from the disease has shown to be a very promising and specific approach for the treatment of SARS-COV-2 infection [104]. This could represent a promising specific approach in the treatment of COVID-19 also on the basis of experience gained in other countries on a limited number of patients [105]. Convalescent plasma therapy also appears to be characterized by a high level of safety, as documented on all occasions in which it has been used in recent years, including COVID-19 itself. However, no randomized controlled trials or controlled non-randomized studies evaluating benefits and harms of convalescent plasma have been completed [106]. The European Commission in its recent Guide to Member States has pointed out that hyperimmune plasma would be a low-risk therapy immediately usable in selected categories of patients and a bridge alternative to be used while waiting for the production of a vaccine or the availability of drugs of proven efficacy [107]. So far, there is general consensus on the importance of combating uncontrolled inflammation, and the resulting ARDS or CSS, caused by this infection.

## 12. Highlights

- COVID-19 is a global pandemic that has currently emerged as one of the most intense and overwhelming viral infection for the humankind to manage.
- As the number of individuals infected with SARS-CoV-2 continues to rise globally, rapid diagnostics at earlier stages, therapeutics, and vaccines will become crucial for the management of the COVID-19 pandemic.
- There is some scientific evidence of efficacy of particular drugs such as antiviral (i.e., RDV), antiparasitic (i.e., hydroxychloroquine), and anti-inflammatory approaches (i.e., tocilizumab) for treatment of COVID-19.
- In the light of the exuberance of the host’s inflammatory response, a potential cause of lung damage and subsequent mortality, the priority should be to identify drugs with potent and specific antiviral, anti-inflammatory, and anticoagulant properties.
- A rational therapy would require careful evaluation of the cytokine profile of selected cohorts of subjects, which include SARS-CoV-2 positive patients with pneumonia or admitted to ICU. COVID-19 clinical evidence has shown that the first seven days of illness are crucial. To initiate therapeutic trials based on the rational use of anti-inflammatory drugs that directly inhibit the synthesis process of inflammatory cytokines including IL-16 and IL-17, would be desirable in the near future.

## Figures and Tables

**Table 1:** Most common pathogens and respiratory diseases that may display symptoms similar to coronavirus diseases (COVID)-19 include the following.

**Virus and Viral Diseases**
Orthomyxovirus (Influenza)
Paramyxovirus (Parainfluenza) PIV-1, -2, -3, and 4
Human metapneumovirus *
Human rhinovirus ^§^
Adenovirus
Coronavirus ^
Enterovirus ^^
Respiratory syncytial virus °
**Bacteria and Bacterial Infections**
Haemophilus influenzae pneumonia
Streptococcus pneumoniae pneumonia
Moraxella catarrhalis pneumonia
Bordetella pertussis and Bordetella parapertussis
Legionella pneumophila °°
Mycoplasma pneumoniae °°
Chlamydia pneumoniae °°

* Human metapneumovirus infection include pneumonia, otitis media, bronchiolitis, exacerbations of asthma, or chronic obstructive pulmonary disease. ^§^ Human rhinoviruses (HRVs) consist of approximately 160 types that cause a wide range of infections, including asymptomatic infections, common colds, and benign or severe lower respiratory illnesses [8]. ^ Four strains of coronavirus including OC43, NL63, HKU1, and 229E usually cause mild symptoms like the common cold or flu [6]. ^^ Enterovirus D68 (also known as EV-D68) is one of more than 100 non-polio enteroviruses known today that causes respiratory diseases [9]. It is found in respiratory secretions such as mucus, saliva, or sputum of an infected person. EV-D68 spreads from person to person when an infected person coughs, sneezes, or touches an object or surface that is then touched by others. ° Respiratory syncytial virus is amongst the most important pathogenic infections of childhood and is associated with significant morbidity and mortality [8]; °° responsible for atypical pneumonia as well as benign upper airway disorders including pharyngodinia, hoarseness, etc. [10].

**Table 2:** Characterization of SARS-CoV-1, Middle Eastern respiratory syndrome (MERS)-CoV, and SARS-CoV-2 spike region, homology with bat coronavirus, and human interaction.

	SARS-CoV-1	MERS-CoV	SARS-CoV-2		
Origin	Guangdong Province, China	Saudi Arabia	Wuhan, China	Rome, Italy	Paris, France
Potential reservoir	Bat *	Bat *	Bat *		
Intermediate host	Palm-civet	Camel/dromedary	Pangolin (to be established yet)		
Final host	Humans	Humans	Humans		
NCBI GenBank No.	AY278741.1	NC_019843.3	MN908947.3	MT077125.1	EPI_ISL_406596 (GISAID No.)
Reference	[37]	[38]	[39]	[33]	[40]
Complete genome length (nt)	29,727	30,119	29,903	29,785	29,853
Spike gene location (nt)	21,492–25,259	21,456–25,517	21,563–25,384	21,507–25,328	21,563–25,384
Spike gene length (nt)	3768	4062	3822		
Spike genomic sequence homology with Bat * #	74.21% (Query cover 97 %)	75.47% (Query cover 8%)	92.89% (Query cover 100%)		92.83% (Query cover 100%)
Spike protein length (aa)	1255	1353	1273		
Spike amino acid sequence homology with Bat * #	76.67% (Query cover 100%)	34.22% (Query cover 87%)	97.41% (Query cover 100%)		97.25% (Query cover 100%)
The predominant receptor	Human angiotensin-converting enzyme-2 (ACE2)	Human dipeptidyl peptidase 4 (DPP4 or CD26)	Human angiotensin-converting enzyme-2 (ACE2)		

Bat *, refers to Bat coronavirus RaTG13 (GenBank accession number: MN996532.1), isolated from *Rhinolophus affinis* bat; #, The BLAST program (https://blast.ncbi.nlm.nih.gov/Blast.cgi) was used to conduct alignment and find sequences of homology and/or variation between the spike region of SARS-CoV-1, MERS-CoV, SARS-CoV-2, and the spike region of the bat coronavirus RaTG13; No., accession number; nt, nucleotides; aa, amino acids.
